# Sex differences in coronary artery disease

**DOI:** 10.1007/s00210-025-04751-2

**Published:** 2025-10-25

**Authors:** Lena Marie Seegers

**Affiliations:** Department of Cardiology and Angiology, Frankfurt University Hospital, Theodor-Stern-Kai 7, 60590 Frankfurt, Germany

**Keywords:** Coronary artery disease, Sex differences, Myocardial infarction, Women’s health, Atherosclerosis

## Abstract

Myocardial infarction is the leading cause of death worldwide in men and in women. Nevertheless, cardiovascular diseases in women are still understudied, underdiagnosed and undertreated leading to poor outcomes and higher mortality rates. Important sex differences exist in coronary artery disease with a higher symptom burden, delayed presentation and treatment of women in the emergency department and more non-obstructive coronary artery disease on angiogram. Sex hormones influence hemostasis and platelet function, and women suffer more from major bleeding complications after coronary interventions. Moreover, cholesterol levels increase with age particularly in women and sex hormones might play an important role in the development and progression of atherosclerosis with an increasing cardiovascular risk for women after menopause. Women also have other unique sex-specific risk factors such as hypertensive disorders of pregnancy that are contributing to an increased risk of myocardial infarction. Therefore, pharmacological therapies regarding primary or secondary prevention of coronary artery disease need to address sex differences to improve female outcome in the future. This review highlights these differences and specific risk factors in women to consider in pharmacological management of coronary artery disease.

## This article is published as part of the special issue sex and gender pharmacology

### Women and coronary artery disease

Coronary artery disease (CAD) is the most common cause of death among women worldwide, affecting 275 million of women each year (2019). Immense efforts to optimize therapies in patients presenting with acute coronary syndromes (ACS) have been devoted in cardiovascular care, developing advanced strategies of initial patient risk assessment, pharmacologic management, and treatment time of the culprit lesion. Despite the overall improve of mortality rate of cardiovascular diseases in the last few decades, women are still more than twice likely to die from myocardial infarction and receive less guideline-directed therapies Clinically women present with a high prevalence of chest pain but, at the same time, also with more accompanying symptoms such as shortness of breath, nausea and fatigue. The simultaneous occurrence of multiple symptoms might contribute to the fact that women with ACS present in emergency department around 30 min later (Vogel et al. 2021; Martinho et al. 2025 Jan 18).

Most of culprit lesions in acute coronary syndrome (ACS) do not present severe stenosis on coronary angiogram (Little et al. 1988; Virmani et al. 2006) Angiography by itself cannot provide details about plaque morphology or visualize thrombus. Intracoronary imaging such as optical coherence tomography (OCT) using near-infrared light enables to identify and characterize rapidly culprit lesions of ACS and diagnose the underlying pathologies of ACS offering an individual specific assessment for interventional cardiologist to decide for the most accurate strategy including quantification of calcium and determination of high-risk plaques. Several studies demonstrated that the pathobiology of ACS is fundamentally different from stable angina pectoris with a greater lipid burden, macrophage infiltration and higher prevalence of high- risk plaques in patients with ACS (Ando et al. 2011; Kato et al. 2012). There are 3 major pathologies known in ACS: plaque rupture, plaque erosion and eruptive calcified nodule. Plaque rupture is, with 60–70% of the cases, the major underlying pathology of ACS and sudden cardiac death (Falk et al. 2013; Higuma et al. 2015). Clinically, patients with plaque rupture often present in the morning hours and have more frequently ST-elevation (STEMI) (Araki et al. 2021). Rupture fibrous caps are commonly associated with a high prevalence of thin cap fibroatheroma (TCFA), a lipid rich atherosclerotic plaque with a large lipid arc and a thin fibrous cap thickness prone to rupture (Virmani et al. 2000; Araki et al. 2022). Macrophage driven destabilization of the plaque results into rupture of the thin fibrous cap and releases highly thrombogenic material of the necrotic lipid core into the vessel lumen with subsequent activation of platelet aggregation and thrombus formation resulting in occlusion of the vessel. Thrombi can be absent due to prior thrombolytic therapies or in cases of old ruptures, and extend thrombus burden can challenge identification of the ruptured plaque. [13.]. Culprit plaques of plaque rupture on OCT have a high prevalence of lipid plaque, TCFA, microvessels and positive remodeling (Higuma et al. 2015; Aguirre et al. 2021).

There are significant sex differences in the development and manifestation of atherosclerosis in the coronary arteries: Despite a higher symptom burden women have less obstructive coronary artery disease. In 5–15% of the cases patients with ACS do not show obstruction on angiogram (Tamis-Holland et al. 2019). The diagnosis myocardial infarction with non-obstructive coronary arteries (MINOCA) is based on the missing obstruction > 50% on angiogram and affects more women. It includes a variation of pathologies such as plaque rupture, erosion or spontaneous coronary artery dissection (SCAD) and is associated with adverse outcomes (Reynolds et al. 2021; Barr et al. 2018) Incomplete understanding of the mechanism of MINOCA makes OCT an essential tool in the management of MINOCA and increases the chances of identifying the culprit lesion and determining the underlying pathology. Indeed, up to 80% of women have atherosclerotic plaque on intracoronary imaging (Smith et al. 2020; Reynolds et al. 2020; Khuddus et al. 2010). Women develop atherosclerosis about 10 years later than men with a step-wise increase after menopause (Lerner and Kannel 1986; Mathur et al. 2015). Coronary plaque characteristics also change by age with more plaque erosion in young women and more inflammatory vulnerable plaque in older women resulting in a higher prevalence of plaque rupture and fatal events (Burke et al. 2001; Seegers et al. 2022; Seegers et al. 2023 Nov). The diversity of symptoms and the dramatic increase of high-risk plaques with age leads to delayed presentation of women with acute coronary syndrome (ACS) in the emergency department and misdiagnosis of myocardial infarction. This contributes to the fact that in-hospital mortality is higher in females with ACS than in males due to longer door-to-balloon times and less instant guideline directed medical therapy. (Bugiardini et al. 2017 Aug 21).

Recently awareness increased about the cardiac impact of sex specific risk factors such as hypertension disorders of pregnancy or early menopause. Black women, women with HDP (hypertensive disorders of pregnancy disorders) and postmenopausal women with obesity are especially at a high risk for cardiovascular mortality (Martin et al. 2025; DeSalvo et al. 2005; Honigberg et al. 2020). Therefore, a complete patient’s anamnesis including gravidity, parity and age of menopause in women should be established in clinical practice. Endogenous estrogen production seems to be protective for cardiovascular risk factors such as hypertension, hyperlipidemia and diabetes. However, postmenopausal substitutional therapy was persistently negative as a preventative therapy for CAD and is not recommended for women in terms of coronary pathologies (Hauge et al. 2022 Jun 14; Honigberg et al. 2019 Dec 24; Mendelsohn and Karas 1999; Mehta et al. 2016). Individual decisions, based on sex-, age- and environment-specific plaque characteristics, including personal PCI strategies and medical treatment should be the future for a tailored management in patients with ACS.

Beside interventional strategies, cornerstones of medical treatment of CAD are lipid lowering therapies and antithrombotic management. Optimal primary and secondary preventive therapy should be provided according to the guidelines regardless of sex. Yet, important sex differences in pharmacological therapies for patients with CAD exist and should be considered.

### Lipid lowering therapies

Hypercholesterolemia is one of the most important modifiable cardiovascular risk factors. While premenopausal women exhibit significantly lower low-density lipoprotein-cholesterol (LDL-C) concentrations compared to age-matched men, a remarkable increase in LDL-C is observed at the onset of menopause (Ambikairajah et al. 2019). Elevated lipid levels are independently associated with increased cardiovascular risk in women. This was confirmed by a large-scale prospective study of women with a 30-year follow-up (Ridker et al. 2024). The study demonstrated a continuous relationship between lower levels of LDL-C and lipoprotein(a) and a reduced incidence of atherosclerotic cardiovascular events. Lipid levels are often underestimated in women, probably due to the step-wise increase after menopause, leading to delayed initiation of lipid lowering medication (Oortmerssen et al. 2025). The European Society of Cardiology Systematic Coronary Risk Evaluation (ESC SCORE) 2 algorithm is a useful tool to identify high risk patients and incorporates sex as a variable in the prediction of the cardiovascular risk and determines LDL-C treatment targets. It can be applied in all patients to estimate the 10-years risk of fatal and non-fatal cardiovascular events. The score includes variables such as age, systolic blood pressure, smoking status and lipid levels. (SCORE2 working group and ESC Cardiovascular risk collaboration 2021 Jul 1). However, sex-specific risk modifiers such as the menopausal transition are still not integrated into the SCORE2 model.

Compared to men, women are less likely to receive lipid lowering therapies and achieve less often LDL target values (Berteotti et al. 2024; Rachamin et al. 2021). Various medications are available for effective cholesterol reduction. Statins are hydroxy-methyl-glutaryl-coenzyme A (HMG CoA) reductase inhibitors and the most available and best studied cholesterol-lowering drug. Intensive statin treatment not only decrease LDL values, but also decelerate the progression of atherosclerosis and stabilize coronary plaques (Kini et al. 2013; Fukumoto et al. 2001). Women are significantly less likely to be prescribed statins and receive more often a subtherapeutic dosage, even after myocardial infarction (Peters et al. 2018 Apr 24). On the other hand, women stop also medication more often due to more side effects reported by females, especially the occurrence of myalgia in those taking statin. (Karalis et al. 2016). Fewer women are included in clinical trials, however, several studies showed that statins are equally effective in women. (Shepherd et al. 2002; Ridker et al. 2008). Women may have higher plasma concentrations of statins due to differences in body composition and metabolism, but dose adjustment is not necessary (Soldin and Mattison 2009). Lower dosage at the beginning with a stepping up after one month might help to avoid adverse reactions in women and increase therapy adherence. Statins and other lipid-lowering agents are contraindicated in pregnancy and during breastfeeding.

Ezetimibe inhibits the absorption of cholesterol in the intestine and it showed improved cardiovascular outcome if added to statin therapy (Cannon et al. 2015). The study included only 24% of women in the trial, but no differences were observed between sexes (Kato et al. 2017 Nov 18). Bempedoic acid can be used in statin intolerant patients and acts like an adenosine triphosphate-citrate lyase inhibitor. The CLEAR Outcome study included 48% women, so bempedoic acid can be used safely in women (Nissen et al. 2023). Proprotein convertase subtilisin/kexin type 9 (PSCK9) inhibitors are monoclonal antibodies to lower LDL-C and showed also the same safety in both sexes with a slightly lower LDL-C reduction in women, but no differences in major cardiovascular events (MACE) (Schwartz et al. 2018; Rivera et al. 2023 Oct 28).

### Antithrombotic management

There are also important differences regarding antithrombotic treatment. Differences in platelet biology between men and women need to be considered in the response of antiplatelet agents. Women have a higher platelet count than men and a higher platelet reactivity (Becker et al. 2006; Breet et al. 2011 Nov). Animal studies revealed effects of sex hormones on platelet activity. Estradiol increases prostacyclin synthesis, a platelet activation inhibitor, and release nitric oxide, which has vasodilative effects and reduce platelet aggregation (O'Sullivan et al. 2001 Jun 15; Nevzati et al. 2015). On the other side, women experience a higher thrombogenic risk especially during pregnancy, oral contraceptive use and hormone replacement therapy.

Mono platelet therapies are part of secondary prevention guidelines in patients with known CAD to avoid platelet accumulation at the atherosclerotic vessel wall containing high thrombogenic material inside the plaque. Aspirin suppresses platelet aggregation by inhibiting cyclooxygenase-1 (COX-1) that promotes platelet aggregation. No significant differences were observed for the safety and efficacy of aspirin for secondary prevention. (Benziger et al. 2024 Sep 1). Dual antiplatelet therapy (DAPT), consisting of aspirin and an inhibitor of the platelet P2Y12 receptor are recommended for all patients undergoing percutaneous coronary intervention (PCI). The greater delay of diagnosis and treatment leads consequently to a later administration of antithrombotic medication in women with ACS. P2Y12 inhibitors are platelet inhibitors binding at the P2Y12 receptor for adenosine diphosphate. Women with ACS receive potent P2Y12 inhibitors such as ticagrelor or prasugrel, recommended by international guidelines after myocardial infarction, less often than men contributing to a higher mortality rate in women (Burgess et al. 2023). The clinical consensus document of the European Association of Percutaneous Cardiovascular Interventions (EAPCI) and the ESC Working Group on Thrombosis provides guidance in the management of antithrombotic therapies in women (Paradies et al. 2025). During PCI women have a higher bleeding risk due to older age, lower body weight and higher prevalence of anemia and renal dysfunction. Femoral access in coronary interventions should be avoided due to higher bleeding risk and vascular complications especially in older women with comorbidities. Renal dysfunction weight adjusted heparin dosing can avoid major periprocedural bleeding complications in women and vulnerable subgroups of women might benefit from a short DAPT management of one up to three months (Chandiramani et al. 2020 Apr 7; Wang et al. 2017 Jan 14; Kunadian et al. 2025 Apr 14). Nonetheless, enrollment of women in trials of antithrombotic drugs needs to be increased for more evidence in the future.

## Conclusion

Despite the significant improve of interventional and pharmacological management over the last few decades, myocardial infarction remains the leading causes of death among women. Women are neither equally represented in clinical trials nor are sex specific risk factors part of the current guidelines for risk stratification of CAD and myocardial infarction. Gaps in undertreatment related to guidelines recommended therapies need to be addressed to improve cardiac health care in women. Better understanding of biochemical mechanisms of sex hormones and their impact on coronary arteries is needed to provide tailored therapies for women and improve their outcome.

## Data Availability

All source data for this work are available upon reasonable request.
